# The Application of Human Figure Drawing as a Supplementary Tool for Depression Screening

**DOI:** 10.3389/fpsyg.2022.865206

**Published:** 2022-06-03

**Authors:** Xuyang Deng, Tiantong Mu, Yu Wang, Yuqi Xie

**Affiliations:** ^1^Center of Mental Health Education, Southeast University, Nanjing, China; ^2^College of Humanities, Southeast University, Nanjing, China

**Keywords:** human figure drawing, depression, screening, college students, projective test, assessment

## Abstract

**Objective:**

Depression is one of the most prevalent mental disorder in college students. The traditional screening method for is psychological measurements or scales, but social desirability can cause students to mask their thoughts, and an auxiliary projective test may be needed. This study was designed to measure the validity of applying human figure drawing (HFD) test as an auxiliary tool for depression screening in this population.

**Methods:**

The HFD test was administered to 113 clinical participants diagnosed with major depressive disorder and 97 healthy college students with self-rating depression scale scores <50. Correlation analysis, chi-square tests, and logistic regression were conducted to identify specific drawing features that associated with depression and could differentiate between the clinical and control subjects. ROC curve was also implemented to evaluate the diagnostic accuracy.

**Results:**

Eleven drawing features were significantly related to depression based on the chi-square test results and seven drawing features were associated with depression based on correlation analysis. After logistic regression by controlling gender and age, three drawing features were associated with depression: shaded eyes, drawing clothes in detail, and drawing other personal belongings. Further, drawing clothes in detail and drawing other personal belongings were two significant variables in ROC curve analysis.

**Conclusion:**

Logistic regression showed that shaded eyes, drawing clothes in detail and drawing other personal belongings were significant drawing features. Individuals with depression will have less energy to put extra effort into drawing and are less likely to have detailed drawings. And the shading of eyes may represent that depressive individuals have a low willingness to communicate and tend to isolate themselves. The results indicated that Human Figure Drawing could be used as an auxiliary tool in college students’ depression screening. Further, the ROC curve analysis showed low discrimination of single drawing features, suggesting that the application of Human Figure Drawing should be considered as a whole instead of focusing on the single drawing feature.

## Introduction

Depressive disorder is currently one of the most common mental health disorders affecting personal psychological well-being. Huang et al. (2019) reported that the lifetime prevalence of any mood disorder in the Chinese population is 7.4%, while it is 6.9% for depressive disorders including major depressive disorders, dysthymic disorder, and not specified depressive disorders. Among Chinese college students, the overall prevalence of depression is 23.8% (Lei et al., 2016). Compared to general population, college students may experience enormous pressure from economic stress, academic demands and interpersonal relationships (Steptoe et al., 2007). The main symptoms of depressive disorders include depressed mood, diminished interest, loss of energy, recurrent thoughts of death, and recurrent suicidal ideation (American Psychiatric Association, 2013). College students are usually young adults, which is the most common age of first psychological illness onset (Kessler et al., 2007). College students in different stages may face varying levels of stress and have different needs. The general risk factors for mental health issues in this population are psychological factors including self-esteem, self-confidence, personality, academic factors, biological factors, lifestyle factors, social factors, and economic factors (Mofatteh, 2021). Currently, Chinese universities use psychometrical measurements to assess and screen the mental health status of college students, such as the Symptoms Checklist-90 (SCL-90), self-rating depression scale (SDS), and University Personality Inventory (UPI). The problem with personality or mental health measurements is that respondents may change their answers to improve social desirability (Edwards, 1957); that is, college students tend to respond to the test contents in a manner expected by society. A fast, effective, and simple projective assessment that masks purpose of the test is needed as a supplementary tool. The use of projective tests methods has been discussed previously. For example, Chen and Xu (2008) found that some drawing features of House-Tree-Person could be used as indexes to diagnosis depression. Further, human figure drawing found to be valid for distinguish children with emotional problems in Brazil samples (Rosa et al., 2019).

Projective test is one way to evaluate an individual’s personality and can be used to explore the depths of unconscious thoughts. They usually employ ambiguous stimuli to evoke responses that may reflect personality characteristics (Gu et al., 2020). Projective tests can be applied to both children and adults. The most famous projective test is inkblot test developed by the Swiss psychiatrist Hermann Rorschach. Unlike a traditional scale or survey measurement, it consists of showing participants 10 inkblots freely and in order, and they are asked to describe what they see and how they perceive it. The responses are recorded and analyzed by psychological interpretation and a complex algorithm, and then researchers or clinicians can understand the individuals’ personality characteristics and emotional functioning. Another classical projective test is the House-Tree-Person (HTP) drawing test developed by psychologist Buck (1948). Originally designed to measure intelligence, it was less used as more targeted tests of intelligence were developed; however, the secondary purposes are as a personality measurement or mental health status screening tool (Polatajko and Kaiserman, 1986). In the beginning, participants are instructed to draw a house, tree, and person on separate pieces of papers. This was the basis of the Kinetic-House-Tree-Person drawing test invented by Burns (1987), where participants would draw all three elements on the same page. With this approach, the interactive relationship of the three elements could be interpreted and analyzed. For example, the relationship between the subject and the family can be seen from the location and distance of the house and person (Burns, 1987).

Human figure drawing (HFD) is another projective test that asks subjects to draw self-portraits. According to Machover’s (1949) theory, HFD is a projection of the artist’s own body image, and Kamano (1960) reports that the results correlated with the actual self the most (*r* = 0.59), while the correlation between HFD and the ideal and least-liked versions of self are 0.35 and 0.36, respectively. Thus, clinicians can infer the individual’s personality traits and emotional state by analyzing the overall portrait and the characteristics of different body parts. There are three main advantages of the HFD. First, the test is convenient, fast, only requires pencil and paper, and can usually be completed within 10 min. Second, it is closely related to the subject’s self-perception of themselves, while other tests such as tree drawing might be too abstract. Third, subjects may not know how their work will be interpreted, so they are less likely to consciously bias their work in specific directions (Groth-Marnat, 2009). The HFD has mostly been applied and researched in the measurement of children’s intelligence (e.g., Scott, 1981; Abell et al., 2001; Willcock et al., 2011), but some researchers have applied it in clinical settings. For example, Holmes and Wiederholt (1982) found that figure size and depression are not correlated since groups of depressed patients, non-depressed patients, and non-depressed hospital employees draw figures close to same size. In contrast to Holmes’ findings, figure drawing height was negatively correlated with depression in a population of psychiatric patients (Lewinsohn, 1964).

As mentioned above, previous work paid more attention to the application of HFD as an intellectual or cognitive assessment for children or adolescents. There is li mited research on the application of HFD in adults and its validity to screen for mental disorders, especially for in the Chinese population. In the present study, correlation analysis and chi-square tests were implemented to find out the association of drawing features and depression, and a classification model for major depressive disorders in young adults was constructed using quantitative indexes of HFD with binary logistic regression. The purpose was to evaluate the feasibility of using HFD as an auxiliary diagnostic tool to screen depressive disorders in Chinese college students and other adult populations.

## Materials and Methods

### Participants

Participants in the clinical group were recruited from one hospital in Nanjing, China. All of them were clinically diagnosed with major depressive disorder by psychiatrists. Their mean [standard deviation (SD)] age was 31.04 (17.57) years; 29.2% (33) were male and 70.8% (80) were female. The mean SDS score of the clinical group was 61.81 (13.99). We screened 133 college students for inclusion in the healthy control group, but 36 were ineligible based on an SDS score >50 indicating symptoms of depressive disorders, so 97 college students were ultimately included in our study. The mean age of the control group was 18.46 (0.87); 70.1% (68) were male, and 29.9% (29) were female. The mean SDS score was 39.28 (6.21). For control group, freshmen and sophomore students who are currently taking mental health education courses were advertised to voluntarily participated this study, they will not receive any class credits or monetary awards. All subjects signed an informed consent form prior to voluntarily participating in the study, which was approved by Nanjing Medical University Ethics Committee. The operation definition of each drawing features was introduced in Table 1.

**TABLE 1:** Operational definition of drawing features.

Type of features	Code	Drawing features	Operational definitions
Structure	A1	Small size	Figure drawing size is smaller than 2/9 of the drawing paper
	A2	Left-sided figures	Main part of drawing within left 1/4 of the drawing paper
Body parts omission	B1	Eyes omitted	Eyes or eyeballs are omitted
	B2	Noses omitted	Nose is omitted
	B3	Hands omitted	At least one hand is omitted
	B4	Feet omitted	At least one foot is omitted
	B5	Eyebrow omitted	Eyebrows are omitted
Shaded and blacken	C1	Shaded hair	Over 80% of hair is shaded or blackened
	C2	Shaded eyebrow	Over 80% of eyebrows is shaded or blackened
	C3	Shaded noses	Over 80% of nose is shaded or blackened
	C4	Shaded mouth	Over 80% of mouth is shaded or blackened
	C5	Shaded eyes	Over 80% of eyes is shaded or blackened
Detailed drawing	D1	Drawing clothes in detail	Style of clothes is recognizable (e.g., T-shirt, dress, skirt, etc.)
	D2	Drawing accessories in detail	Texture of accessories (e.g., watches, bracelets, etc.) are recognizable
	D3	Drawing limbs in detail	Details of hands and feet (e.g., Joints, nails, and shoe styles) are recognizable
	D4	Drawing at least two facial features	Facial features are recognizable and are not constructed by single lines
	D5	Drawing other personal belongings	Personal belongings are recognizable (e.g., bags, guitar, etc.)

### Research Tools

#### Human Figure Drawing

Human Figure Drawing test is a projective test used to assess the personality state and cognitive function of individuals. Each participant was required to draw a complete person with a pencil on A4 paper. There was no specific time limit, but participants usually finished within 10 min. The features of the HFD were based on the Koppitz emotional indicators (Koppitz, 1968, 1984); if one feature was present, the score was 1, if absent, the score was 0. Feature evaluation was independently carried out by two psychology graduate students who completed professional drawing evaluation training. Before the assessments started, the examiner explained the process and trained the assistants on the operational definition of HFD characteristics. The operation definition of each drawing features was introduced in Table 1.

#### Self-Rating Depression Scale

The SDS was used to measure all participants’ depression level. Originally developed by Zung (1965), it contains 20 items that are rated on a 4-point Likert scale. Higher scores represent more severe depressive symptoms. Based on Chinese norms, adults with SDS scores ranges of 53–62 have symptoms of mild depression, 63–72 have symptoms of moderate depression, and scores ≥73 indicate severe depression (Duan and Sheng, 2012). In this study, we used the general Chinese population cutoff of 50.

### Data Analysis

Based on the emotional indicators of HFD, drawing features of HFD were considered as independent variables (Machover, 1953; Koppitz, 1968, 1984), the appearance and absence of a certain painting feature was coded as 1 and 0, respectively. According to previous studies on HFD, the size, location, lack, and emphasis of body parts can all reflect different emotional conditions. Kappa coefficient was performed to assess the inter-rater reliability. Correlation analysis was performed to find out the association between drawing features with gender, age and depression. Chi-square tests were performed to determine which indexes showed significant differences between the healthy and clinical groups. Binary logistic regression was conducted to determine the relationship of specific drawing features and depression after controlling gender and age. All significant variables from chi-square test (*p* < 0.1) were entered into the logistic regression model, and then backward elimination method was used to simplify the model. Statistically significant variables from logistic regression analysis were included into Receiver operating characteristic curve (ROC) analysis to evaluate the diagnostic accuracy. All analyses were performed with SPSS v24.0 software (IBM Corp., Armonk, NY, United States) and *P* < 0.05 was considered significant.

## Results

Firstly, kappa coefficients were performed to assess the inter-rater reliability. The Kappa coefficient for all drawing features reached 0.6, which represents the substantial agreement (McHugh, 2012). The kappa coefficient for drawing features ranged from 0.752 (Drawing clothes in detail) to 1.000 (Feet Omitted).

The results of correlation of 17 drawing features with age, gender and SDS scores is depicted in Table 2. Five drawing features were associated with age, eyebrows omitted (*r* = 0.374, *p* < 0.001), shaded hair (*r* = −0.155, *p* = 0.024), shaded eyebrow (*r* = −0.181, *p* = 0.008), drawing at least two facial features (*r* = −0.154, *p* = 0.026) and drawing other personal belongings (*r* = −0.198, *p* = 0.004). Two drawing features were associated with gender, shaded noses (*r* = −0.201, *p* = 0.003) and drawing other personal belongings (*r* = −0.146, *p* = 0.034). Seven associated features were associated with SDS scores, individuals with higher SDS scores will more likely to have features of small drawing size (*r* = 0.159, *p* = 0.021), and less likely to have features of shaded hair (*r* = −0.168, *p* = 0.015), shaded eyebrow (*r* = −0.141, *p* = 0.041), shaded noses (*r* = −0.157, *p* = 0.023), drawing clothes in detail (*r* = −0.183, *p* = 0.008), drawing limbs in detail (*r* = −0.141, *p* = 0.042) and drawing other personal belongings (*r* = −0.219, *p* = 0.001).

**TABLE 2:** Correlation between SDS total scores, gender, and age with drawing features.

Code	Features	SDS total scores		Gender		Age	
		*r*	*p*	*r*	*p*	*r*	*p*
A1	Small size	0.159	0.021	0.109	0.115	0.122	0.078
A2	Left-sided figures	-0.061	0.383	0.010	0.886	0.003	0.969
B1	Eyes omitted	0.087	0.211	-0.034	0.624	0.087	0.207
B2	Noses omitted	0.085	0.221	-0.007	0.922	-0.128	0.064
B3	Hands omitted	0.108	0.120	-0.010	0.884	-0.025	0.715
B4	Feet omitted	0.117	0.092	0.049	0.484	-0.098	0.157
B5	Eyebrow omitted	0.023	0.743	0.130	0.060	0.374	<0.001
C1	Shaded hair	−0.168	0.015	-0.057	0.407	-0.155	0.024
C2	Shaded eyebrow	-0.141	0.041	-0.087	0.207	-0.181	0.008
C3	Shaded noses	-0.157	0.023	-0.201	0.003	-0.117	0.092
C4	Shaded mouth	0.081	0.245	0.032	0.648	0.013	0.849
C5	Shaded eyes	0.020	0.774	0.113	0.101	-0.087	0.210
D1	Drawing clothes in detail	-0.183	0.008	0.083	0.231	-0.115	0.096
D2	Drawing accessories in detail	-0.048	0.489	0.114	0.100	-0.135	0.051
D3	Drawing limbs in detail	-0.141	0.042	-0.068	0.327	0.107	0.121
D4	Drawing at least two facial features	-0.130	0.059	-0.111	0.109	-0.154	0.026
D5	Drawing other personal belongings	-0.219	0.001	−0.146	0.034	-0.198	0.004

**FIGURE 1:**
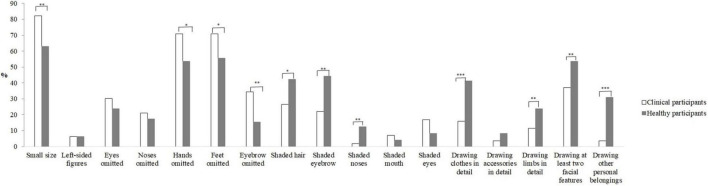
Frequency of drawing features and sex in clinical participants and healthy participants.

The chi-square test results of drawing features are shown in Figure 1 and Table 3. Eleven drawing features were significantly different between the healthy and clinical groups. Compared to healthy subjects, drawings by people with depression were more likely to have the following features: small drawing size (χ^2^ = 10.060, *p* = 0.002), hands omitted (χ^2^ = 6.605, *p* = 0.010), and feet omitted (χ^2^ = 5.172, *p* = 0.023), eyebrow omitted (χ^2^ = 9.915, *p* = 0.002). Depressive individuals were less likely to draw shaded hair (χ^2^ = 5.763, *p* = 0.016), shaded eyebrows, (χ^2^ = 11.755, *p* = 0.001), shaded noses (χ^2^ = 9.428, *p* = 0.002), drawing clothes in detail (χ^2^ = 16.723, *p* < 0.001), drawing limbs in detail (χ^2^ = 5.476, *p* = 0.019), drawing at least two facial features (χ^2^ = 5.705, *p* = 0.017), and drawing other personal belongings (χ^2^ = 28.854, *p* < 0.001).

**TABLE 3:** Chi-square test results for drawing feature frequencies.

Code	Features	Clinical participants (*N* = 113)	%	Healthy participants (*N* = 97)	%	Chi-square	*p*
		*n*		*n*			
A1	Small size	93	82.3	61	62.9	10.060	0.002
A2	Left-sided figures	7	6.2	6	6.2	0.000	0.998
B1	Eyes omitted	34	30.1	23	23.7	1.073	0.300
B2	Noses omitted	24	21.2	17	17.5	0.458	0.499
B3	Hands omitted	80	70.8	52	53.6	6.605	0.010
B4	Feet omitted	80	70.8	54	55.7	5.172	0.023
B5	Eyebrow omitted	39	34.5	15	15.5	9.915	0.002
C1	Shaded hair	30	26.5	41	42.3	5.763	0.016
C2	Shaded eyebrow	25	22.1	43	44.3	11.755	0.001
C3	Shaded noses	2	1.8	12	12.4	9.428	0.002
C4	Shaded mouth	8	7.1	4	4.1	0.846	0.358
C5	Shaded eyes	19	16.8	8	8.2	3.419	0.064
D1	Drawing clothes in detail	18	15.9	40	41.2	16.723	<0.001
D2	Drawing accessories in detail	4	3.5	8	8.2	2.147	0.143
D3	Drawing limbs in detail	13	11.5	23	23.7	5.476	0.019
D4	Drawing at least two facial features	42	37.2	52	53.6	5.705	0.017
D5	Drawing other personal belongings	4	3.5	30	30.9	28.854	<0.001

Table 4 shows the results of logistic regression analyses. After controlling age and gender, depression individuals were significantly less likely had drawing features of detailed clothing (*p* < 0.001) and other personal belongings (*p* = 0.005). Individuals with depression were significantly more likely to have drawing features of shaded eyes (*p* = 0.034). The Nagelkerke *R*^2^ is 0.641. The *R*^2^ of null model with control variables of gender and age is 0.503, the change of *R*^2^ is 0.138.

**TABLE 4:** Logistic regression analyses of drawing features.

Code	Drawing features	*B*	*S.E.*	*p*	OR	95% CI for OR	
						Lower	Upper
C5	Shaded eyes (1)	−1.281	0.606	0.034	0.278	0.085	0.911
D1	Drawing clothes in detail (1)	1.924	0.511	<0.001	6.849	2.516	18.643
D5	Drawing other personal belongings (1)	1.836	0.648	0.005	6.274	1.762	22.336

*CI, confidence interval; OR, odds ratio. Age and gender were added into model for adjustments.*

**FIGURE 2:**
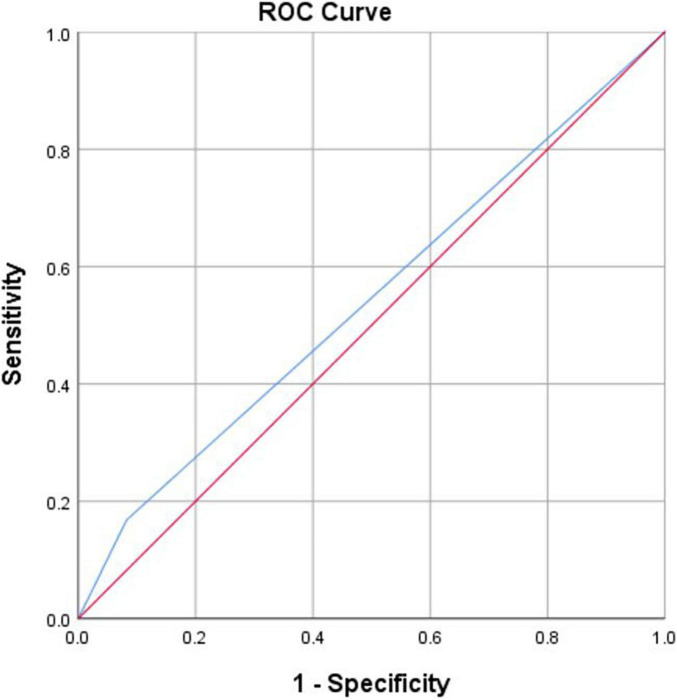
ROC curve of drawing features positively associated with depression.

**FIGURE 3:**
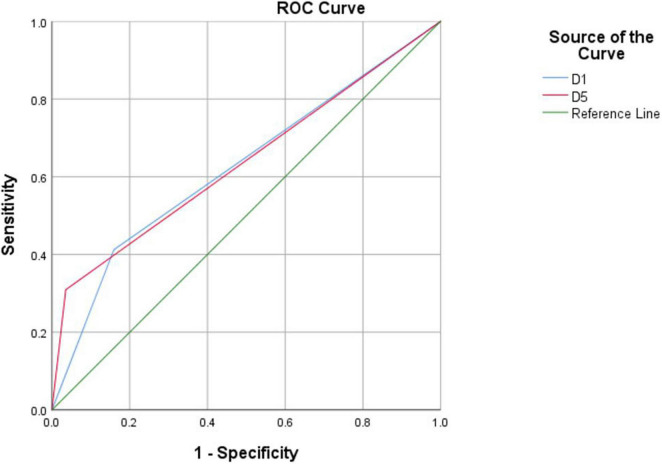
ROC curve of drawing features negatively associated with depression.

An analysis of the AUC, septicity, and sensitivity are shown in Tables 5, 6. The Figures 2, 3 shows the ROC curve of drawing features. The results indicate that drawing clothes in detail (*p* = 0.002) and drawing other personal belongings (*p* = 0.001) were significant variables to discriminate depression, with AUCs of 0.627 and 0.637, respectively.

**TABLE 5:** Results of ROC curve analysis of drawing features positively associated with depression.

Code	Independent variables	AUC	*S.E.*	*p*	95% CI		Sensitivity	Specificity
					Lower	Upper		
C5	Shaded eyes	0.543	0.040	0.285	0.465	0.621	0.168	0.918

*AUC, area under curve; S.E., standard error; CI, confidence interval.*

**TABLE 6:** Results of ROC curve analysis of drawing features negatively associated with depression.

Code	Independent variables	AUC	*S.E.*	*p*	95% CI		Sensitivity	Specificity
					Lower	Upper		
D1	Drawing clothes in detail	0.627	0.039	0.002	0.550	0.703	0.412	0.841
D5	Drawing other personal belongings	0.637	0.039	0.001	0.560	0.714	0.309	0.965

*AUC, area under curve; S.E., standard error; CI, confidence interval.*

## Discussion

Drawing is one of the most ancient human activities, and it is an easy and effective way to illustrate emotions. As a non-verbal projective test, the HFD could be a helpful tool to assess an individual’s personal development, attitudes, and personality characteristics (Koppitz, 1984). Many researchers reported the good inter-rater reliability of HFD (Swensen, 1968). There are multiple emotional indicators of the HFD test that can be classified into five categories: impulsivity, insecurity, anxiety, shyness, and aggression (Koppitz, 1983). Ryan-Wenger (2001) used a summed score of emotional indicators in children’s emotional problems analyses, but this approach has been critiqued, and some emotional indicators in the HFD better reflect emotional states. Cultural differences should also be considered. For example, small drawing size is more prevalent in the Mexican children than American children (Koppitz and De Moreau, 1968). Little attention has been paid to the validity of emotional indicators in the Chinese population. Here we assessed the HFD as a potential auxiliary tool to screen depression in Chinese college students by examining which emotional indicators could significantly differentiate between healthy and depressive samples. The results of the chi-square test and correlation analysis revealed that depression rates were significantly different depending on drawing size, omission of body parts, shading of facial features, and level of detail. However, after controlling the gender and age, only three drawing features were significant, shaded eyes, drawing clothes in detail, and drawing other personal belongings.

According to Koppitz’s (1968, 1984) interpretation, small drawing size could be explained by shyness, insecurity, lack of self-confidence, and withdrawal. Individuals may draw tiny figures to enhance one’s sense of security. In our study, individuals with depression were more likely to have a small drawing size by both the chi-square test and correlation analysis. Conversely, Sandman et al. (1968) found no significant relationship between drawing size and depression scores. The difference between Koppitz’s theory and Sandman’s founding could be due to the fact that Sandman only assessed psychiatric patients. The omission of body parts such as hands and feet can be understood as intense anxiety and insecurity regarding the missing parts, and individuals may feel a sense of guilt. The chi-square and correlation analysis indicated that omission of body parts was significantly correlated with depression, with subjects in the clinical group less likely to draw feet, hands, and eyebrows. However, after controlling gender and age, the omission of body parts and small drawing size were no longer significant, which may represent that omission of body and small drawing size were more influenced by gender and age, instead of depression. Shading of facial features represents the emphasis on specific parts. Koppitz (1984) proposed that face shading reflects severe anxiety toward future events or being troubled and in a worried state. However, we found that facial feature shading was less common in the clinical group. Others have reported different findings regarding hair and head shading. According to an HFD literature review, 4 studies agree with Koppitz’s traditional explanation of shading, 5 reported the opposite findings, and 10 reported non-significant results (Handler and Reyher, 1965). There are some explanations for these discrepancies. Handler and Reyher (1964) demonstrated that most clinical subjects will finish the figures with a minimum effort; in this case, shading, reinforcement, and erasure may reflect coping with anxiety. Our result of correlation analysis and chi-square test showed that shading of hair, eyebrow, and nose was negatively related to depression, individuals with depression will have more shading in those facial features. Conversely, shading of eyes was positively correlated with depression based on the results of logistic regression. The eyes are special facial features, they are primary communication organs since infanthood. Shaded eyes or eyes covered by dark glasses, according to Koppitz’s theory, may represent social isolation and refusal to face the real world. Detailed drawings with more supplementary features other than the person indicate a positive mental state because the subject has a better understanding of one’s own image and sufficient energy to draw it. Our study further promoted the ideas of Handler and Reyher (1965), in Chinese culture, individuals with depression will be less likely to have detailed drawings and more likely to shade eyes in the drawing. It might represent that depressive individuals will have less energy to complete the drawing with details, or they are not willing to do it. Further, individuals with the drawing feature of shaded eyes may represent a low willingness to communicate with others and tend to isolate themselves.

The ROC curve analysis showed unsatisfied results, only two features, drawing clothes in detail and drawing other personal belongings were significant with low AUCs. Previously, some researchers studied the discrimination of projective tests based on ROC curve analysis. For example, Kim and Chung (2021) developed the drawing tests for predicting depression among breast cancer patients, they reported an AUC of 0.82, a sensitivity of 0.85, and a specificity of 0.64. They evaluated ROC with summed scores of 23 formative elements, including the number of colors used, degree of integration, closure status, etc. We do not assess the summed scores of HFD, instead, we evaluate the diagnostic accuracy through a single drawing feature, this may explain why we did not achieve the optimal results.

Drawing is also a form of art therapy, which can achieve good outcomes. For example, Saunders and Saunders (2000) reported that adolescents with problematic behaviors demonstrated significant improvements. Even simple art production can reduce the participant’s negative mood and anxiety (Bell and Robbins, 2007), suggesting that the application of HFD in the college settings could therefore also play a “treatment” role.

## Conclusion

The current study is exploratory, aiming to measure the validity of the application of HFD as a supplementary tool in college depression screening to overcome the shortages of traditional scale measurements in Chinese colleges where students may lie or mask their answers due to social desirability. The chi-square test and correlation analysis results of HFD tests were significant. Small drawing size and body parts omission were positively associated with depression, while shading of facial features, except for eyes, and detailed drawing showed negative associations. Among shading of facial features, shading of eyes showed the opposite result, which was positively related to depression. Although we identified 11 significantly different emotional indicators by chi-square test, after controlling gender and age, only three indicators, shading of eyes, drawing clothes in detail, and drawing other personal belongings in detail, were associated with depression, which corresponds with a previous study (Engle and Suppes, 1970). Individuals with depression often lack energy, so even small tasks take extra effort, and they have slower thinking. As a result, they are less likely to put extra effort into the drawing. The shading of eyes may represent social isolation and low willingness to communicate with others. Further, the results of the ROC curve analysis showed that single drawing features showed poor discriminability. Therefore, the HFD should only serve as an auxiliary role in mental disorders screening, and the human figure drawing should be considered as a whole instead of focusing on a single indicator. Compared to house-tree-person tests, the operational definition of HFD tests is easier, and the rater does not need extensive training.

## Limitation

There are several shortcomings of this work. First, the healthy subjects that we recruited were a convenience sample from an institute of technology, and the majority were male since we did not control the gender equivalence. Secondly, the cross-sectional design precluded assessment of the HFD’s test-retest reliability. Thirdly, the sample size was small. Future studies could expand the sample size to explore the application of HFD as a screening method for depression. Finally, the sensitivity of HFD is relatively low, represents the low “true positive” rate of HFD features. As aforementioned above, the use of single drawing feature for depression diagnosis is not recommended. The future study could further develop a score system for HFD to obtain the better diagnostic accuracy. Another possible future research direction is that assessing the individuals’ self-stigma and social desirability of mental health issues to find out whether HFD is a satisfactorily supplementary tool to screen depression for individuals who have high level of self-stigma and social desirability.

## Data Availability Statement

The raw data supporting the conclusions of this article will be made available by the authors, without undue reservation.

## Ethics Statement

The studies involving human participants were reviewed and approved by the Nanjing Medical University. The patients/participants provided their written informed consent to participate in this study.

## Author Contributions

XD conceived the study, analyzed the data, and drafted the manuscript. TM and YX recruited participants and developed the instruments. YW edited the draft and conducted the literature review. All authors contributed to the article and approved the submitted version.

## Conflict of Interest

The authors declare that the research was conducted in the absence of any commercial or financial relationships that could be construed as a potential conflict of interest.

## Publisher’s Note

All claims expressed in this article are solely those of the authors and do not necessarily represent those of their affiliated organizations, or those of the publisher, the editors and the reviewers. Any product that may be evaluated in this article, or claim that may be made by its manufacturer, is not guaranteed or endorsed by the publisher.
